# Vaccination of mice with hybrid protein containing Exotoxin S and PcrV with adjuvants alum and MPL protects *Pseudomonas aeruginosa* infections

**DOI:** 10.1038/s41598-022-05157-3

**Published:** 2022-01-25

**Authors:** Mohammad Reza Asadi Karam, Farzad Badmasti, Khadijeh Ahmadi, Mehri Habibi

**Affiliations:** 1 Department of Molecular Biology, Pasteur Institute of Iran, Tehran, Iran; 2 Department of Bacteriology, Pasteur Institute of Iran, Tehran, Iran; 3 Infectious and Tropical Diseases Research Center, Hormozgan Health Institute, Hormozgan University of Medical Sciences, Bandar Abbas, Iran

**Keywords:** Immunology, Microbiology, Molecular biology, Diseases, Pathogenesis

## Abstract

*Pseudomonas aeruginosa* as a common pathogen causing urinary tract infections (UTIs) has been resistant to different antibiotics and developing an effective vaccine can be an alternative strategy. In the present study, the immunogenicity and protection efficacy of formulations composed of a hybrid protein composed of *P. aeruginosa* V-antigen (PcrV) and exoenzyme S (ExoS) with alum and MPL were evaluated. The hybrid protein could increase the specific systemic and mucosal immune responses, as well as cellular responses as compared with control groups. Combining of alum or MPL adjuvant with the hybrid protein significantly improved the levels of IgG1, serum IgA, mucosal IgG, and IL-17 as compared to the ExoS.PcrV alone. After bladder challenge with a *P. aeruginosa* strain, the bacterial loads of bladder and kidneys were significantly decreased in mice received ExoS.PcrV admixed with alum and ExoS.PcrV admixed with MPL than controls. The present study indicated that immunization of mice with a hybrid protein composed of ExoS and PcrV could induce multifactorial immune responses and opsonize the bacteria and decrease the viable bacterial cells. Because *P*. *aeruginosa* have caused therapeutic challenges worldwide, our study proposed ExoS.PcrV + alum and ExoS.PcrV + MPL as promising candidates for the prevention of infections caused by *P*. *aeruginosa*.

## Introduction

Urinary tract infection (UTI) is a common community- and nosocomial-acquired infection in the world that infects approximately half of women and 12% of men in their lifetimes. In addition, a quarter of primary UTIs will result in recurrent infections between 6 and 12 months which antibiotic therapy of these infections is usually ineffective^1–3^. Although, cystitis as a common form of UTI is treatable by antibiotics, but complicated forms of UTI including acute pyelonephritis, bacteremia or septicemia are serious and may be fatal for patients^3^.

Nowadays, *Pseudomonas aeruginosa* strains regarded as one of the serious nosocomial pathogens worldwide. *P. aeruginosa* has acquired the ability to cause infection in different organs especially respiratory and urinary tract^4,5^. Furthermore, *P. aeruginosa* have acquired the versatility in different environments to cause different infections in men and women^5^. Susceptible humans especially those with immunocompromised immunity, burns, organ transplantation, and cystic fibrosis (CF) suffer more from infection with *P. aeruginosa*^6^.

Currently, the effective way for treatment of UTI pathogens such as *P. aeruginosa* is antibiotics. But, the misuse and frequent use of antibiotics resulted in the antibiotic resistance especially resistance to trimethoprim–sulfamethoxazole (TMP–SMX), cephalosporines, and quinolones as the first- and second-line antibiotic therapies against UTIs^3,7^. In addition, antibiotic therapy increases the medical costs, decreases quality of life of patients, may effect on commensal microbiota of patients and can increase the chance of secondary infections^3,8^. Thus, developing of a preventive vaccine can be an effective and cost-effective alternative to antibiotic therapy against UTI pathogens especially *P. aeruginosa*.

The previous studies about developing of a vaccine against *P. aeruginosa* usually focused on designing of candidates based on fimbrial components, flagella, lipopolysaccharide (LPS), outer membrane proteins, components of secretion systems, and exoenzymes^5,9^. In addition, some of the candidates showed promising results in animal models and have been tested in clinical trial phase I-III. Despite notable advancement in development of effective vaccines against *P. aeruginosa*, there is no approved vaccine as a universal vaccine for use in the world^4,9^.

Type III secretion system (T3SS) in gram negative bacteria especially *P. aeruginosa* is regarded as an important virulence characteristic that performs different functions for virulence of the pathogen^10,11^. T3SS is composed of different parts that *P. aeruginosa* V-antigen (PcrV) is located in the end of needle structure of T3SS. PcrV constitutes in assembly of translocons of T3SS and directs the translocation of effector enzymes including exoenzymes T (ExoT), ExoS, ExoU, and ExoY into the target cells^12,13^.

Exoenzyme S (ExoS) is known as an important cytotoxin in pathogenesis of *P. aeruginosa* strains in different infections^14^. N-terminal domain of ExoS as a GTPase-activating protein (GAP domain) and C-terminal domain as an adenosine diphosphate ribosyl-transferase domain (ADPRT-domain) cause apoptosis of host cells and also contribute in colonization of *P. aeruginosa* in the patients^15,16^.

As mentioned, antigens such as PcrV have been evaluated as promising vaccine candidates against *P. aeruginosa* infections. According to the criteria of an ideal vaccine, in the previous study, we constructed a hybrid protein composed of the full-length PcrV protein and C-terminal domain of ExoS from *P. aeruginosa* by bioinformatics studies^17^. Then, expression and purification of the recombinant protein were done in a prokaryotic expression system^17^. In the present study, we aimed to evaluate the immunogenicity and protective potential of the vaccine target with and without human adjuvants alum and MPL in animal model.

## Results

### Humoral response to the vaccine candidates

**Figure 1:**
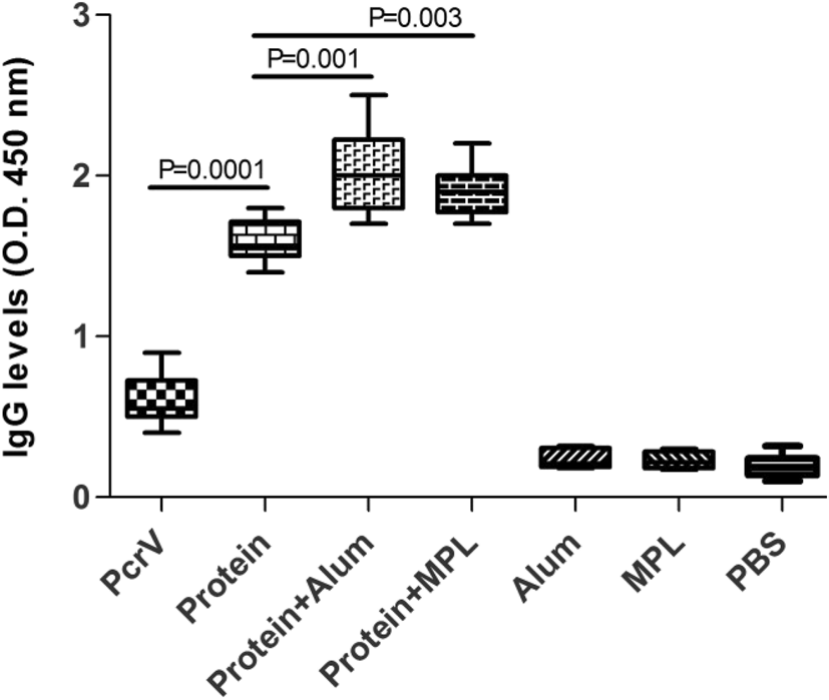
Measurement of serum IgG responses. Mice were vaccinated subcutaneously three times with PcrV alone, hybrid protein alone (protein), hybrid protein admixed with alum (Protein + Alum), hybrid protein admixed with MPL (Protein + MPL). The control groups were given Alum, MPL, and PBS. Blood samples were collected and anti-hybrid protein IgG levels were measured by ELISA. Significant values are shown with P value. Data are shown as mean ± SD of three independent experiments at dilution 1:800.

We used subcutaneous route of administration to elicit a significant humoral response against the designed vaccine target alone or formulated with alum and MPL adjuvants. A significant increase of specific IgG antibody was developed against the vaccine candidate as compared to the control groups (p < 0.001) that MPL and alum significantly increased the IgG responses (p = 0.003 and p = 0.001, respectively) (Fig. 1). In addition, a significant level of IgG response was developed against the hybrid protein as compared to the PcrV alone group (p = 0.0001). In comparison, no significant difference could be observed in the IgG responses between the mice received protein admixed with alum and protein admixed with MPL (p > 0.05) (Fig. 1).

### Isotypes antibody responses to the vaccine candidates

**Figure 2:**
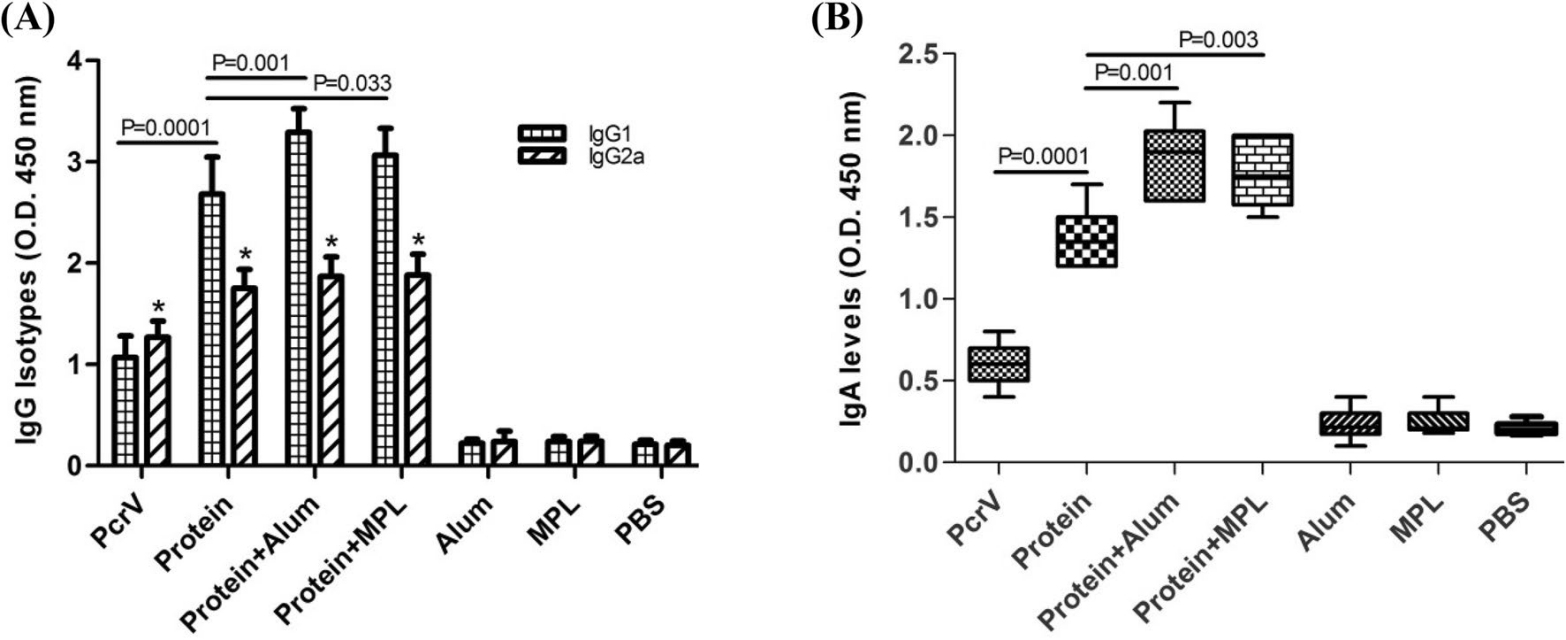
Analysis of isotypes antibodies responses in serum. The levels of IgG1 and IgG2a isotypes, as well as IgA responses were detected after the last immunization. (**A**) IgG1 and IgG2a, and (**B**) IgA responses. The significant levels between different groups are presented with P value. In addition, asterisks show the statstical difference in IgG2a level between the vaccinated groups with the control groups (p < 0.05). The experiments were repeated three times. The results are mean ± S.D. from 10 mice per groups at dilution 1:800.

In the next stage, the levels of isotypes IgG1, IgG2a, and also IgG1/IgG2a ratio were determined in the mice groups to find the direction of immune responses toward Th1 or Th2. After the last vaccine dose, vaccinated mice groups including PcrV alone, hybrid protein ExoS.PcrV, hybrid protein + alum, and hybrid protein + MPL produced a significant level of both IgG1 and IgG2a as compared to the control groups (p < 0.01) (Fig. 2A). Combining of alum or MPL adjuvant with the hybrid protein significantly enhanced anti-protein IgG1 level as compared to the ExoS.PcrV alone (p = 0.001 and p = 0.033, respectively). In contrary, addition of alum or MPL to ExoS.PcrV had no significant effect in increasing the level of IgG2a than with ExoS.PcrV alone (p > 0.05) (Fig. 2A). In addition, mice received hybrid protein ExoS.PcrV could develop more IgG1 level than with mice vaccinated with PcrV alone (p = 0.0001). We also could not find a significant difference in inducing the IgG1 and IgG2a levels between the mice immunized with protein + alum and protein + MPL (p > 0.05) (Fig. 2A). The serum anti-protein IgA level in the ExoS.PcrV group was higher than PcrV alone (p = 0.0001) and control groups (p < 0.001) and mixing the hybrid protein with alum or MPL adjuvant induced a significant level of anti-protein IgA as compared with hybrid protein alone (p = 0.001 and p = 0.003, respectively) (Fig. 2B).

### Mucosal antibody responses to the vaccine candidates

**Figure 3:**
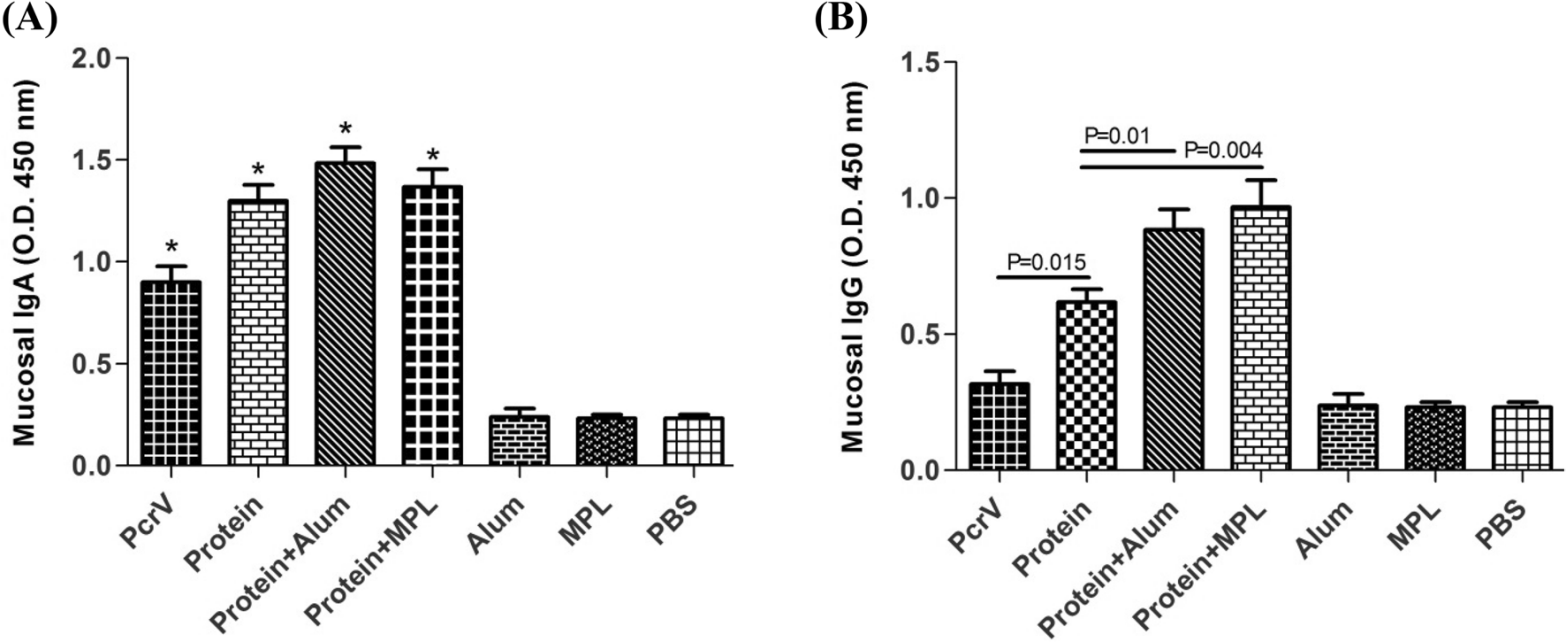
Detection of mucosal responses in the urine. After the last immunization, (**A**) mucosal IgA and (**B**) mucosal IgG levels were detected by ELISA in the urine samples. The significant difference in IgA levels between the immunized mice groups with control mice is shown with single asterisks. Also, the significant differences in IgG levels are shown with P value. The results are the average of three independent experiments. Data were shown as the mean ± SD (16 mice/ group) at dilution 1:2 of the urine.

Then, the levels of anti-ExoS.PcrV IgG and IgA were determined in the urine samples of immunized mice to evaluate the mucosal responses. Interestingly, vaccination of mice with hybrid protein ExoS.PcrV alone by subcutaneous route showed the ability to significantly increase the anti-ExoS.PcrV mucosal IgG and IgA than with mice received PBS, alum or MPL (p < 0.01) (Fig. 3). Furthermore, it was found that ExoS.PcrV developed more anti-ExoS.PcrV IgG as compared to the PcrV alone (p = 0.015). Similar to serum IgG responses, alum and MPL showed their potential to significantly enhance the anti-ExoS.PcrV mucosal IgG than with hybrid protein alone (p = 0.01 and p = 0.004, respectively). Furthermore, it was found that there were no significant differences in inducing IgG or IgA responses between the mice received combinations protein + alum and protein + MPL (p > 0.05) (Fig. 3).

### Opsonophagocytic killing activity (OPK)

**Figure 4:**
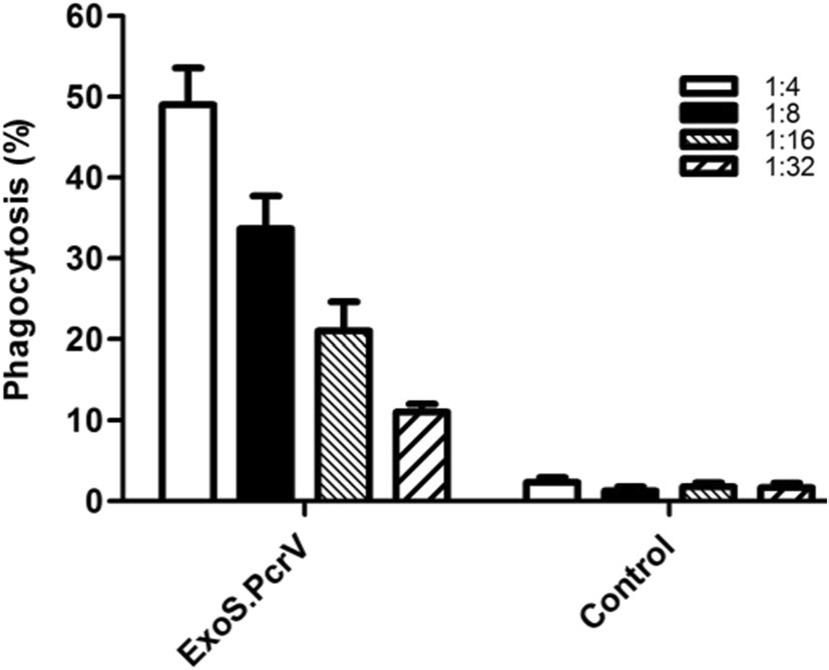
The opsonic killing activity of anti-ExoS.PcrV against *P. aeruginosa* strain PAO1. The *P. aeruginosa* strain PAO1 was incubated with four dilutions of anti-ExoS.PcrV, mice macrophage and rabbit complement. A significant opsonic killing activity was observed when anti-ExoS.PcrV was used as compared to the control group (49% vs. 2.33%). Data are the mean ± S.D. from three independent experiments.

In the present study, in vitro  bioactivity of antibody developed against hybrid protein ExoS.PcrV was determined by OPK assay with *P. aeruginosa* strain PAO1. In this assay, addition of anti-ExoS.PcrV to the complex of macrophages and complement source significantly increased the phagocytosis of *P. aeruginosa* strain PAO1 as compared to the control group (p < 0.001) (Fig. 4). In the control group (PBS), only 2.33% opsonic killing activity was observed. At 1:4 dilution of anti-ExoS.PcrV, the number of viable bacteria cells reduced over 47% as compared to the control group. At 1:4 dilution, the highest opsonic activity of the hybrid protein was observed that was significantly higher than the other serum dilutions (p < 0.01) (Fig. 4).

### Cytokine responses to the vaccine candidates

**Figure 5:**
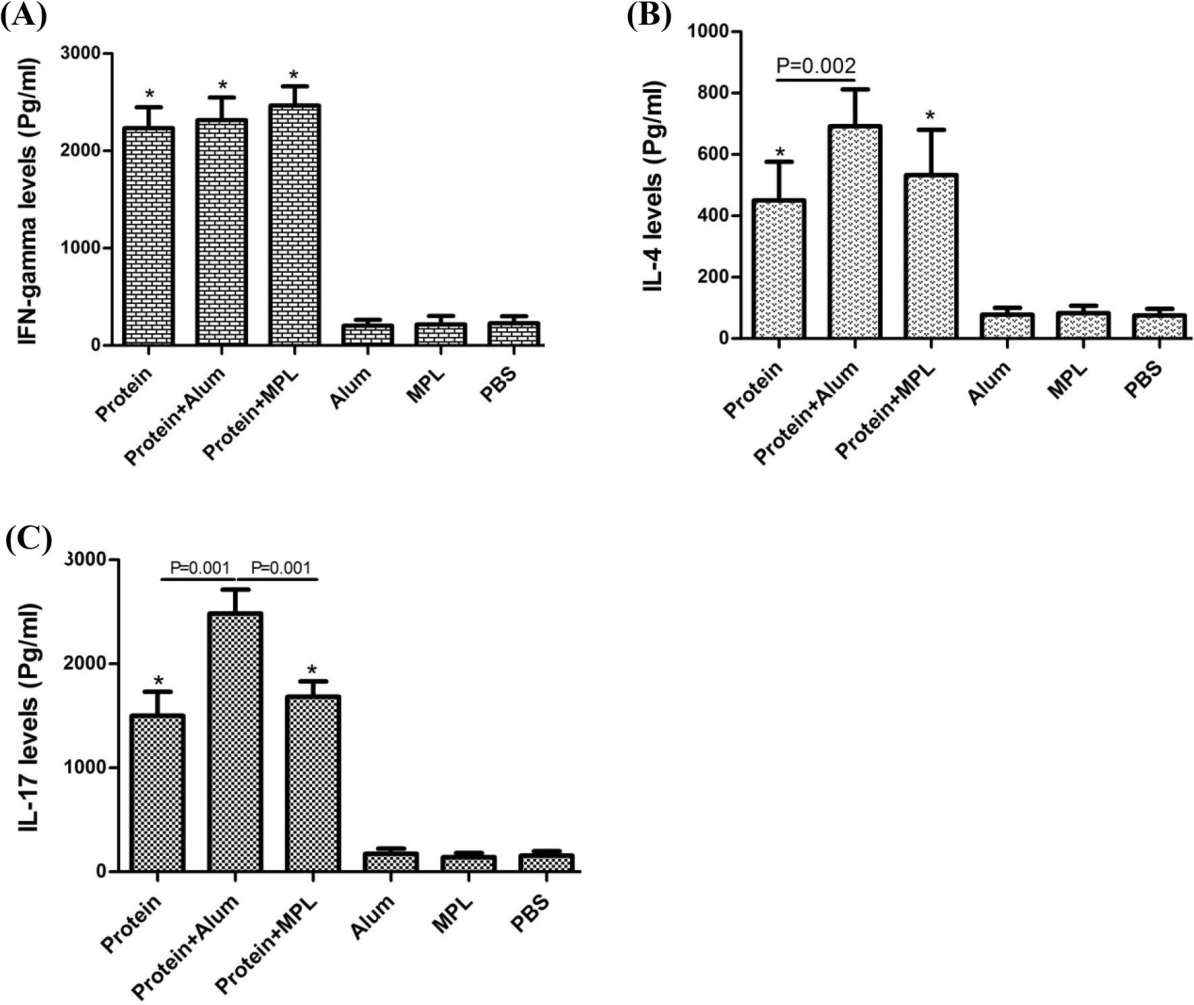
Production of cytokines from the immunized mice. Two weeks after the last administration, splenocytes of mice were collected and cultured with recombinant ExoS.PcrV for 72 h to determine the levels of (**A**) IFN-γ, (**B**) IL-4, and (**C**) IL-17. The significant differences between the immunized groups and control groups are shown with single asterisks. Furthermore, some significant differences are shown with P value. Data are the mean stimulation index ± S.D. of six mice per group from three independent experiments.

Measurement of cytokine levels of IFN-γ and IL-4 as indicators Th1 and Th2 responses, as well as IL-17 was performed in the stimulated splenocytes. Mice which received formulations ExoS.PcrV alone, ExoS.PcrV + alum, and ExoS.PcrV + MPL secreted more level of IFN-γ, IL-4, and IL-17 than mice which received PBS, alum or MPL (p < 0.01) (Fig. 5). It was found that IL-17 secretion was significantly higher in the splenocytes related to ExoS.PcrV + alum and ExoS.PcrV + MPL groups than splenocytes from ExoS.PcrV group (p = 0.001) (Fig. 5C). In addition, combining alum adjuvant with ExoS.PcrV significantly enhanced the level of IL-4 than ExoS.PcrV alone, whereas there was no significant difference between the IL-4 levels from ExoS.PcrV + MPL and ExoS.PcrV groups (p > 0.05) (Fig. 5B). We also found that addition of alum or MPL adjuvant to ExoS.PcrV could not significantly increase the secretion of IFN-γ as compared to ExoS.PcrV alone (p > 0.05) (Fig. 5A).

### Measurement of protective effect of the vaccine candidates

**Figure 6:**
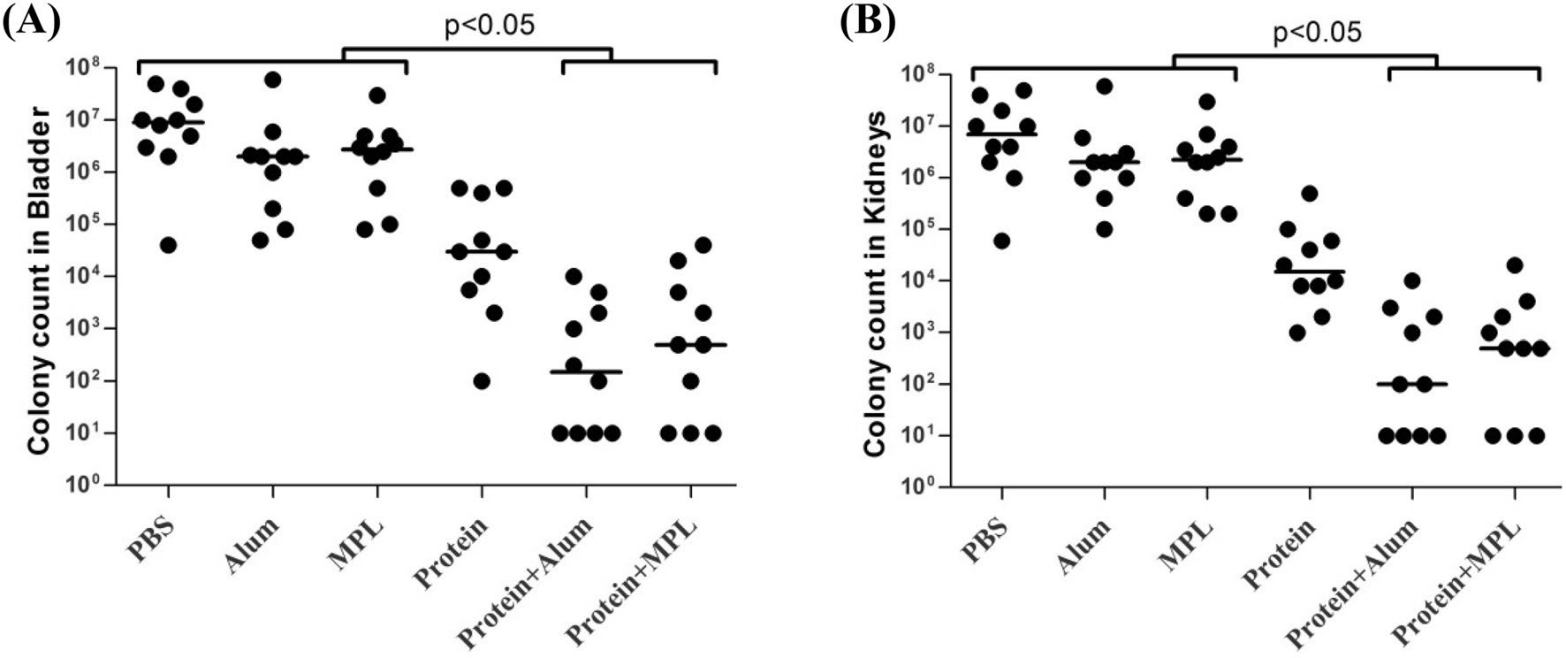
Protective efficacy of the vaccine combinations in a challenge model. Mice were immunized with vaccine formulations and challenged with *P. aeruginosa* strain by transurethral route. Thereafter, the bacterial burdens in the (**A**) bladder and (**B**) kidneys of the mice were counted after 2 days. Symbols show the bacterial count of each mice group with median of the colonization number. Statistical significance of the differences between mice groups is shown by brackets with P value.

The bladder challenge was performed to determine the protection level of the vaccine combinations in the mice model. According to our results, the bacterial loads of bladder and kidneys were significantly reduced in ExoS.PcrV + alum and ExoS.PcrV + MPL groups than in PBS, alum, and MPL groups (P < 0.05). But, vaccination of mice with ExoS.PcrV alone did not significantly improve the protection level in the bladder and kidneys of mice compared with control groups (p > 0.05) (Fig. 6). Among the mice immunized with combinations ExoS.PcrV + alum and ExoS.PcrV + MPL, the mice received mixture of ExoS.PcrV with alum indicated the best level of protection in both bladder and kidneys, however the difference in protection level between these groups was not statistically significant (p > 0.05) (Fig. 6).

## Discussion

Nowadays, *Pseudomonas aeruginosa* by natural or acquiring resistance to different classes of antibiotics become a major problem in the healthcare systems of the countries^4^. According to a report in 2017 in the United States, approximately 32,600 cases of Multi-drug resistance (MDR) *P. aeruginosa* infections was occurred in hospitalized patients that resulted in 2700 deaths and imposed approximately $757 million costs^18^.

Thus, different researchers have focused on developing of an effective and safe vaccine against these infections^9^. In this way, it seems that blocking of the compartments of type III secretion system (T3SS) such as PcrV and exoenzymes can be a promising strategy to prevent or treat the *P. aeruginosa* infections^9,11^. The antibody vaccines based on T3SS compartments have been studied in preclinical and clinical phases. MEDI3902 was developed as an antibody vaccine against PcrV from *P. aeruginosa* that could protect animals against *P. aeruginosa* infections^9^. The other murine monoclonal antibody against PcrV, mAB166, which was developed by Sawa et al. could inhibit translocation of the T3SS toxin^11,19,20^. The Phase II clinical trials of PEGylated anti-PcrV antibody, KB001, indicated reduction of neutrophils and prevented the developing of pneumonia in patients^10^. In addition, two studies demonstrated that polyclonal anti-PcrV IgG collected from human sera protected *P. aeruginosa* infection in animal model^21,22^.

One of the major obstacles in providing of a vaccine against *P. aeruginosa* infections is covering all of the serotypes. The majority of vaccine candidates for example vaccines based on the LPS or outer membrane proteins (OMPs) could not largely be effective against different serotypes of *P. aeruginosa*^9^. In addition, the complex pathogenicity of *P. aeruginosa* and the diverse functions of the virulence factors are the other major obstacles in the development of vaccine against *P. aeruginosa*^19^.

The other studies indicated the important roles of PcrV and ExoS in pathogenesis of *P. aeruginosa* infections and the conservation of PcrV and ExoS in *P. aeruginosa* from clinical samples has been demonstrated in several studies^9,14,23–25^. According to these criteria, maybe the use of these factors in a hybrid protein could prevent the *P. aeruginosa* infections and cover different *P. aeruginosa* serotypes. One of the other reasons to select ExoS component in the hybrid protein was the possible adjuvant properties of C-terminal domain of ExoS (ADPRT domain)^26^. In the previous study, we could demonstrate that ADPRT domain of ExoS in hybrid protein ExoS.PcrV strongly interacted with Toll-like receptor-2 (TLR2) on the immune cells and this interaction could induce pro-inflammatory cytokines, as well as acquired immune responses^17^. In the present study, it was found that mice vaccinated with hybrid protein ExoS.PcrV could develop significantly more systemic (IgG, IgG1, and IgA) and mucosal humoral responses (IgG) than mice received PcrV alone. The possible adjuvant properties of ExoS in hybrid protein ExoS.PcrV could be a mechanism for inducing the anti-PcrV responses. In fact, after interaction of ExoS in the hybrid protein with TLR on antigen presenting cells (APCs) such as dendritic cells (DCs), the entire hybrid protein could be taken up by the APCs to increase the levels of the immune response^27^. In the other study, the auto-adjuvant property of OprI antigen of *P. aeruginosa* was reported by interaction with the TLR2/TLR4 in different vaccine candidates^28–30^. In addition, the auto-adjuvant property of flagellin A and B of *P. aeruginosa* was observed in vaccine candidate OprF–OprI–flagellins^31^.

Normally, nasal route of immunization is considered as the first choice for vaccination against mucosal infections such as UTI and pneumonia^32^. In the current study, although subcutaneous route of immunization was applied to evaluate the efficacy of the hybrid protein, in addition to the serum humoral responses, a noticeable mucosal response was developed in the immunized mice with the vaccine combinations. This may be explained by the transfer of serum IgG from blood into the mucosal systems or production of mucosal responses by plasma cells in the urinary tract^33^. This finding suggests that subcutaneous route as a systemic route of vaccination can be used as an alternative route for vaccination against UTI caused by *P. aeruginosa.* In another study, the efficacy of the intramuscular route of immunization was demonstrated as an alternative route for efficient immunization against pneumonia caused by *P. aeruginosa*^32^*.*

The other studies demonstrated that alum mainly induces Th2 type immune responses and this adjuvant is used to induce the humoral response (Th2)^34^, whereas MPL indicated the ability to induce both humoral and cellular responses^35–38^. MPL as a derivative of LPS from *Salmonella minnesota* activates APCs by interaction with TLR4 and induces the immune responses^35,39^. In the present study, alum induced a significant increase in both anti-protein IgG1 and IL-4 levels but a smaller enhance in IgG2a and IFN-γ levels compared with protein alone, which indicates Th2-biased immune responses. Furthermore, MPL by inducing a marked IgG1, decrease in the IFN-γ/IL-4 ratio, and also increase the IgG1/IgG2a ratio as compared to hybrid protein alone showed tendency to direct the anti-protein responses toward humoral (Th2) responses. In accordance with our results, another study found that immunization of mice with recombinant PcrV alone resulted in significantly higher anti-PcrV IgG1 and addition of alum to PcrV significantly improved anti-PcrV IgG1 than with PcrV alone; whereas there was no statistically significant difference in inducing IgG2a in mice vaccinated with PcrV + alum and PcrV alone (P > 0.05)^40^. In contrary to these findings, in one study, intranasal administration of PcrV admixed with alum could not increase the levels of anti-PcrV in the mice and failed to protect them against *P. aeruginosa*, suggesting that alum in this vaccine could not induce effective humoral immunity^21^.

In the challenge assay, the protection level of mice immunized with adjuvant-free hybrid protein was approximately 10^4^ CFU vs. 10^7^ CFU for control groups, suggesting that vaccination of mice with the hybrid protein alone was able to provide partial protection. Further, increased levels of protection were observed in bladder and kidneys of mice when an adjuvant was used with the hybrid protein, which suggest the importance of adjuvant for improving the protective efficacy of vaccines. In another study, Yang et al.^32^ reported that a fusion protein consisting of PcrV-OprI-Hcp (POH) could protect immunized mice against experimental bacterial challenge and the use of adjuvants alum, Freund’s, AS04, MF59, and AlPO_4_ could enhance the efficacy of protection in a pneumonia and burn model. Hamaoka et al.^40^ also indicated that the numbers of bacteria in the lungs of immunized mice with PcrV + alum were significantly lower than mice received PcrV alone.

In our study, a mixed of Th1, Th2, and IL-17 responses were produced in the serum, urine, and spleens of the immunized mice, thus the induced immune response to vaccination could be the reason for the effective protection against experimental infection. However, it not defined which type of immune response was pivotal in the protection. Comparison of the induced responses by the hybrid protein alone with control groups suggests that humoral and cellular responses could protect mice vaccinated with the hybrid protein alone. In addition, the significant opsonic killing activity of antibodies developed against hybrid protein ExoS.PcrV could led to decrease in the bacterial load in the bladder and kidneys. The better protection of mice vaccinated with the hybrid protein admixed with alum or MPL could be the result of enhanced humoral (IgG, IgG1, and mucosal IgG), IL-4, as well as IL-17 production. In another study, results of Yang et al.^32^ suggest that humoral response is critical in protection of mice immunized with a fusion composed of PcrV-OprI-Hcp1 that was in accordance with the other reports that anti-OprI and PcrV were critical for protection against *P*. *aeruginosa*^19,41^. Although, Yang et al.^32^ reported that cellular immune response was also contributed to the protection induced by the fusion protein. In this regard, von Specht et al.^42^ and also Weimer et al.^31^ indicated that the level of IgG induced against antigens of *P. aeruginosa* was correlated with the level of protection. Interestingly, the findings of Weimer et al.^31^ indicated that protection of mice after intramuscular immunization with fusion protein OprF–OprI–flagellin of *P. aeruginosa* was not dependent on the mucosal antigen specific IgA. In contrary to these findings, some studies have reported a Th1 response; independent of humoral immunity resulted in effective protection against *P*. *aeruginosa* infections^19,43,44^. In several studies the important role of some components of innate immunity, such as macrophage and neutrophil has been indicated for complete protection against *P*. *aeruginosa* infections^45,46^.

One of the striking findings of the study was induction a significant level of IL-17 in immunized mice especially in mice vaccinated with protein + alum that could improve the level of protection of the immunized mice. Nowadays, the important role of IL-17 as a neutrophil-recruiting cytokine in inducing effective protection against infections such as *P*. *aeruginosa* is reported. For example, recombinant PopB-PcrH and an OprL mutant vaccine induced a strong IL-17 response and conferred protection against several clinical *P*. *aeruginosa* isolates^47,48^. In another study, immunized mice with PopB antigen protected against pneumonia caused by *P*. *aeruginosa* in an IL-17-dependent manner without significant antibody-production^48^.

In conclusion, the present study indicated that active immunization with a novel hybrid protein composed of ExoS and PcrV from *P*. *aeruginosa* induced multifactorial immune responses in mice. In addition, alum and MPL as human safe adjuvants improved the immunogenicity and protection efficacy of the novel hybrid protein in a bladder challenge model. Because *P*. *aeruginosa* have caused therapeutic challenges worldwide, our findings proposed two promising vaccine candidates including ExoS.PcrV + alum and ExoS.PcrV + MPL for the prevention of *P*. *aeruginosa* infections. Additional optimization of these vaccine candidates especially evaluation the protective efficacy of the candidates against different serotypes of *P*. *aeruginosa* is under way.

## Methods

### Ethics statement

In the present study, animal studies were performed according to the ethical safety guidelines of Pasteur Institute of Iran and confirmed by the Ethical Committee of the Pasteur Institute of Iran under Ethical Number: IR.PII.REC.1398.014. All experiments were performed in accordance with relevant guidelines and regulations and we explicitly confirm that the studies were approved by the Ethical Committee of the Pasteur Institute of Iran. In addition, we confirm that all methods are reported in accordance with ARRIVE guidelines.

### Designation of the construct and protein expression

We recently designed a hybrid gene composed of *exoS* and *pcrV* genes from the genome of *P. aeruginosa* strain PA01, according to the bioinformatics studies^17^. According to the study, hybrid gene ExoS.PcrV was constructed by Overlapped PCR method and expressed in pET28a-BL21 (DE3) expression system. After confirmation of its expression, purification of the recombinant protein was performed by Nickel resin column and quantified by Bradford assay^17^.

### Mice immunization

In the present study, Balb/C mice were provided from the Pasteur institute of Iran to perform animal studies. Four groups of mice (n = 16 per group) on days 0, 14, and 28 were given subcutaneously vaccine combinations including PcrV alone (50 µg/mice), ExoS.PcrV alone (50 µg/mice), mixture of ExoS.PcrV with alum, and mixture of ExoS.PcrV with MPL. In addition, control mice groups received alum alone (100 µl/mice), MPL alone (100 µl/mice) and PBS (100 µl/mice). For preparation of hybrid protein + alum, ExoS.PcrV protein and aluminum hydroxide were mixed before immunization and gently rotated for 2 h to help binding. After centrifugation, the concentration of unabsorbed protein in the supernatant was determined using the BCA assay kit. Our results showed binding efficacy 98% for alum to the hybrid protein. Then, the prepared mixture of the hybrid protein and alum was used for immunization of mice. In addition, MPL adjuvant (Sigma, USA) was prepared according to the manufacturers' instruction. Briefly, 1 ml of 0.2% triethylamine was added to 1 mg of lyophilized powder of MPL, vortexed until complete solubilization, and then sonicated. Finally, the MPL solution (1 mg/ml) applied at the final concentration of 10 μg in the vaccine preparation.

### Determination of anti-ExoS.PcrV IgG levels

Fourteen days after the final vaccine dose (day 42), peripheral blood and also urine samples were collected from the mice to measure the induced antibody responses by Enzyme-linked immunosorbent assay (ELISA) in the vaccinated mice groups. Briefly, 96-well ELISA plates (Greiner, Germany) were coated with purified protein ExoS.PcrV (10 µg/ml) in coating buffer (0.1 mM NaHCO3, pH = 7.4). Thereafter, the coated plates were blocked with bovine serum albumin (BSA; Sigma) in PBS. Different serum and urine dilutions of the mice groups and goat anti-mouse IgG (Sigma, USA) were applied as primary and secondary antibodies, respectively. Then, H_2_SO_4_ stopped the reactions to read the absorbance at O.D. 450 nm after addition of tetramethylbenzidine (TMB) substrate. Measurement of isotypes antibody (IgG1, IgG2a, and IgA) was also performed by a similar protocol related to total IgG. But, after addition of mice serum, secondary antibodies specific for each isotypes were applied to detect the isotypes antibodies.

### Opsonophagocytosis assay

The opsonophagocytosis assay was performed according to the method of Faezi et al.^49^ with strain PAO1. Macrophages were collected from the naive mice and the blood from a baby rabbit (Pasteur Institute of Iran, Karaj, Iran) used as a complement source. In addition, the serum collected from the immunized mice with formulation ExoS.PcrV was heated at 56 °C for 30 min and used for Opsonophagocytic killing (OPK) activity. Briefly, for OPK assay, a bacterial suspension of *P. aeruginosa* strain PAO1 (2 × 10^9^ cells per well) was incubated with different dilutions of mice sera (1:4 to 1:32) for 60 min. After washing, the mice macrophages (1 × 10^6^ cells per ml) and serum of baby rabbit were added to the complex. The opsonic killing activity of the sera was compared to the control (PBS) group. The percentage of opsonic activity of the sera was calculated as follows:

$${\text{Opsonophagocytosis}}\;\left( \% \right) = \left( {{1} - \left( {{\text{CFU}}\;{\text{ of}}\;{\text{ immune}}\;{\text{ serum}}/{\text{CFU }}\;{\text{of}}\;{\text{ pre - immune}}\;{\text{serum}}} \right)} \right) \times {1}00$$

### Determination of cytokines

Detection of cytokines induced by the vaccine formulations in the immunized mice was done by an optimized protocol. Briefly, the spleens of each mice group (n = 6/group) were recovered, lysed and re-suspended. The suspensions were cultured at 3 × 10^5^ cells/well and incubated with purified protein ExoS.PcrV (10 µg/ml) for 72 h at incubator 37 °C. Then, the collected supernatants were used to measure the levels of cytokines IFN-γ, IL-4 and IL-17 using ELISA cytokine detection kit (R&D systems, Minneapolis, MN).

### Infectious challenge with *P. aeruginosa*

In the present study, *P. aeruginosa* strain PAO1 was used to perform the bladder challenge assay, according to an optimized procedure. Briefly, a suspension containing 10^8^ CFU/ml of the cultured bacteria was prepared in PBS and adjusted with culture and absorbance at O.D. 600 nm. Then, 30 µl of the suspensions were inoculated transurethrally into the bladder of anesthetized mice. After 2 days, the bladders and kidneys tissues of the challenged mice (n = 10 per group) were collected, homogenized and cultured in agar medium to determine the colony count of the bacteria.

### Statistical analysis

In the present study, data were expressed as mean ± S.D. Graphs were depicted with GraphPad (GraphPad Software Inc., San Diego, CA). Data of the study were analyzed by Student’s t-test and One-way analysis of variance (ANOVA) with Tukey’s multiple-comparison test for multiple mice groups. A value of P < 0.05 was considered statistically significant.
